# The impact of heat and impaired kidney function on productivity of Guatemalan sugarcane workers

**DOI:** 10.1371/journal.pone.0205181

**Published:** 2018-10-05

**Authors:** Miranda Dally, Jaime Butler-Dawson, Lyndsay Krisher, Andrew Monaghan, David Weitzenkamp, Cecilia Sorensen, Richard J. Johnson, Elizabeth J. Carlton, Claudia Asensio, Liliana Tenney, Lee S. Newman

**Affiliations:** 1 Center for Health, Work & Environment, Colorado School of Public Health, University of Colorado Anschutz Medical Campus, Aurora, Colorado, United States of America; 2 Colorado Consortium on Climate Change and Human Health, University of Colorado Denver, Aurora, Colorado, United States of America; 3 National Center for Atmospheric Research, Boulder, Colorado, United States of America; 4 Department of Biostatistics and Informatics, Colorado School of Public Health, University of Colorado Anschutz Medical Campus, Aurora, Colorado, United States of America; 5 Department of Emergency Medicine, School of Medicine, University of Colorado Anschutz Medical Campus, Aurora, Colorado, United States of America; 6 Division of Renal Diseases and Hypertension, School of Medicine, University of Colorado Anschutz Medical Campus, Aurora, Colorado, United States of America; 7 Department of Environmental and Occupational Health, Colorado School of Public Health, University of Colorado Anschutz Medical Campus, Aurora, Colorado, United States of America; 8 Pantaleon, Guatemala City, Guatemala; 9 Division of Pulmonary Sciences and Critical Care Medicine, School of Medicine, University of Colorado Anschutz Medical Campus, Aurora, Colorado, United States of America; 10 Department of Epidemiology, Colorado School of Public Health, University of Colorado Anschutz Medical Campus, Aurora, Colorado, United States of America; Massachusetts General Hospital, UNITED STATES

## Abstract

**Background:**

Climate change has implications for human health and productivity. Models suggest that heat extremes affect worker health, reduce labor capacity, and commodity supply. Chronic health conditions are on the rise internationally. However there is a paucity of direct empirical evidence relating increasing temperatures to both agricultural worker health and productivity.

**Methods and findings:**

We evaluated the relationship between temperature exposure, kidney function, and two measures of productivity—tons of commodity produced and job attrition, of 4,095 Guatemalan sugarcane cutters over a 6-month harvest. We used distributed lag non-linear models to evaluate associations between wet bulb globe temperature (WBGT) and productivity of workers with normal or impaired kidney function. The cumulative effect of exposure to a max WBGT of 34°C was 1.16 tons (95% CI: -2.87, 0.54) less sugarcane cut over the next five days by workers with impaired kidney function, compared to exposure to 29°C. Impaired kidney function was associated with premature workforce attrition. Workers starting the harvest season with impaired kidney function were more than twice as likely to leave employment (HR: 2.92, 95% CI: 1.88, 4.32).

**Conclusions:**

Heat extremes may be associated with loss of agricultural worker productivity and employment, especially among those with impaired kidney function. Agricultural workers who develop health conditions, such as kidney disease, are particularly vulnerable in the face of climate change and increasing heat extremes. The resultant loss of employment and productivity has significant implications for global commodity supplies.

## Introduction

One of the most pressing challenges facing the world is the increasing impact of climate change on human health and productivity [1,2]. Rising temperatures will reduce labor capacity [3]. The decline in world economic productivity will disproportionately affect developing countries located in warm, tropical climates [4]. Decreased production in these major food producing regions will threaten food supplies worldwide [5] at a time when global demand for agricultural production in developing countries will rise by an estimated 77% [6].

There is emerging evidence that the adverse effects of increasing global temperatures may already be affecting worker productivity [7]. Multiple studies have demonstrated how worker productivity declines as heat increases [4,8–10]. However only a few studies have examined the meteorological effect on productivity in an agricultural setting, and those have focused on only healthy workers [7,11]. Less is known about how heat’s impact on agricultural worker health [12] contributes to productivity loss, although theoretical models predict negative effects of increasing temperatures on both worker health and performance [13].

For agricultural workers, occupational exposure to hot environments has been identified as a major climate related concern [12,14]. For example, the pandemic of Chronic Kidney Disease of unknown origin (CKDu) among sugarcane cutters and other workers in Central America [15,16], Sri Lanka [17], Egypt [18], and India [18] has been attributed, in part, to heat stress and resulting recurrent dehydration [2,12,19]. A small pilot study has suggested a relationship between agricultural worker productivity and reduced kidney function [20], but no study to date has examined how heat exposure and kidney function are associated with drop out from the workforce, despite the potential implications of attrition on agricultural production. There is an urgent need to understand the implications of heat exposure in agricultural workers internationally, given the global increase in disease and disability, including kidney disease [21].

To assess the relationship between worker health, heat exposure, and productivity, we evaluated daily, averaged agricultural worker productivity of 4,095 Guatemalan sugarcane cutters over a six-month harvest season. We identified those who met the definition of impaired kidney function at the start of the harvest season, and related these findings to local heat conditions. We hypothesized that there would be a direct inverse relationship between temperature exposure, measured as the wet bulb globe temperature (WBGT), and the amount of sugarcane produced. Furthermore, we hypothesized that the productivity of workers with impaired kidney function would be more highly affected by temperature exposure. Finally, we hypothesized that workers with impaired kidney function would be more likely to leave the workforce prior to the end of the six-month harvest season. To our knowledge, this is the first study to directly investigate the relationship between kidney function, heat, and agricultural productivity.

## Methods

### Population and study design

To explore the relationship between kidney insufficiency, increased heat exposure, and agricultural productivity, we examined a cohort of sugarcane cutters who were employed by a large agribusiness in southwest Guatemala. Guatemala is a recognized hotspot for chronic kidney disease [22], has a large agrarian workforce [23], and is at risk of experiencing more extreme heat days due to climate change [24].

**Fig 1:**
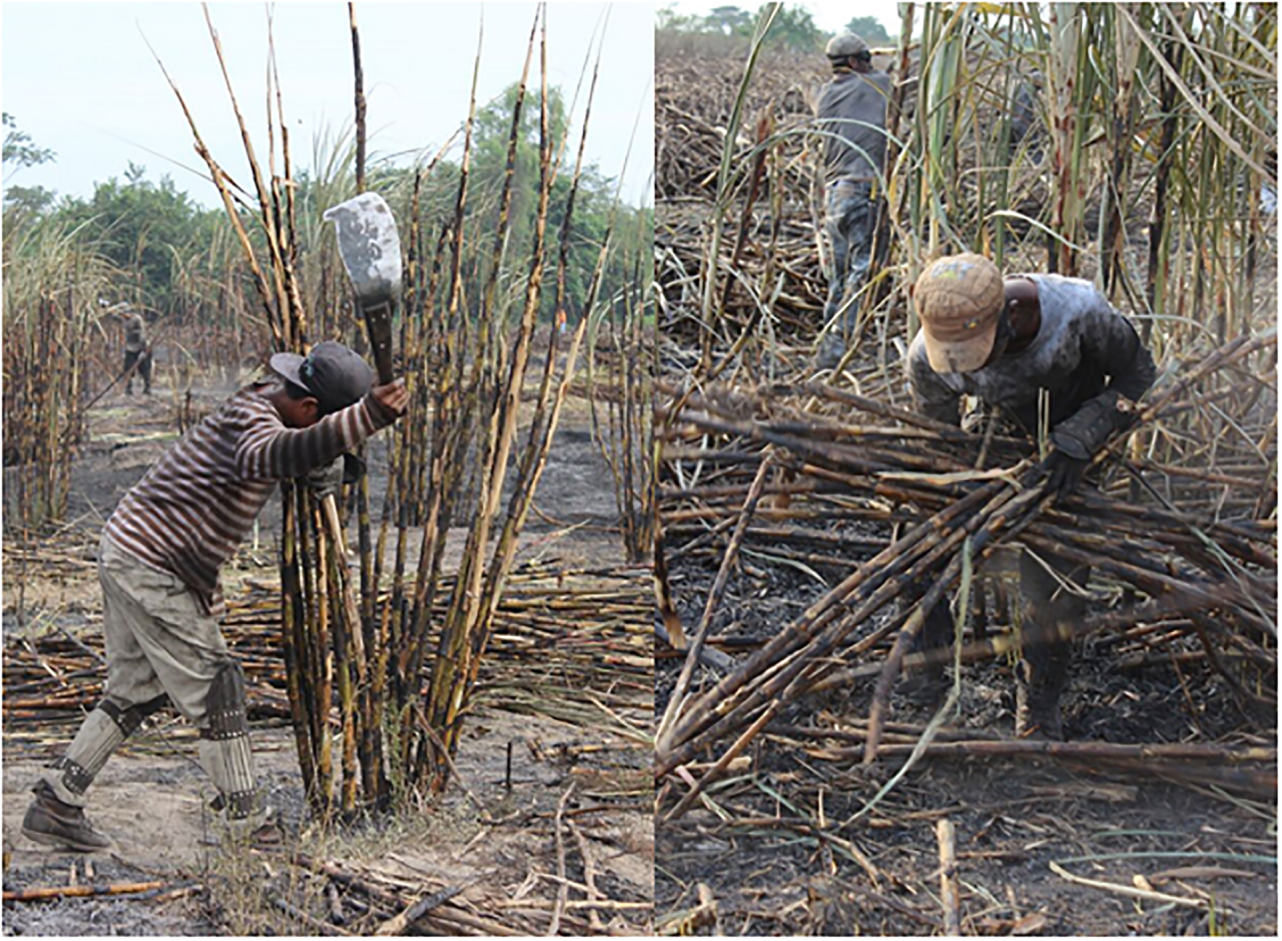
Demonstration of sugarcane cutting in practice. Photo credit: Amanda Walker. (Left) Sugarcane cutter using a machete to cut sugarcane. (Right) Cut sugarcane being collected and stacked.

After a one-week acclimatization period in November, during which employees work shorter days and cut fewer tons of sugarcane, workers typically work eight-hours during a ten-hour shift in six-day blocks before receiving one rest day. Cutting sugarcane is considered very heavy work involving swinging a machete to cut the stalk a few centimeters above ground level, followed by lifting, trimming and stacking the cane (Fig 1).

Workers receive a base wage regardless of the amount of sugarcane harvested. Since 2009, the agribusiness has been promoting hydration, rest, and shade use for the cane cutters, aligned with U.S. Occupational Safety and Health Administration (OSHA) recommendations [25]. This includes instructing cane cutters to drink 16 L of water and 2.5 L of an electrolyte solution (composition per liter: 4.6 g NaCl, 34 g carbohydrates (26 g sucrose) and 2 g KCl) and take three 20-minute breaks and one 60-minute lunch break in the provided shade during the work shift. Water and electrolyte solution are provided daily by the agribusiness.

Our population was drawn from a cohort of workers employed by the agribusiness for the November 2015 to May 2016 harvest season. Per company protocol, the employer screened 5,138 sugarcane workers from August to November 2015, prior to the start of the six-month sugarcane harvest. This pre-employment screening included a 1) medical exam (blood pressure, heart rate, height, and weight), 2) survey that collected information about each individual’s demographics, lifestyle behaviors, and occupational and medical history, and 3) venipuncture to examine serum creatinine, a measure of kidney function. Company policy was to either not hire or place workers in lighter duty jobs if their pre-employment serum creatinine was 1.45 mg/dL or more. In 2015, 107 workers (2.5%) were excluded due to high creatinine. To be included in our analyses, workers must have worked the first week of the harvest, had pre-employment serum creatinine measurements, and had completed the pre-employment survey. Per company protocol, all workers must have been at least 18 years of age at the start of the harvest. Ethics review and approval for our evaluation of these data were received from the Colorado Multiple Institution Review Board (COMIRB). This was a secondary evaluation of de-identified data historically collected by the agribusiness and deemed non-human subjects research. Consent was not required.

#### Measures of productivity

We considered two measures of productivity: average daily tons cut per work day and worker attrition. We calculated average daily tons cut per work day using the average of all workers for each day of the harvest. We calculated this measure for all workers combined, as well as stratified on kidney function. Worker attrition was defined if a worker did not work through all 28 weeks of the harvest. The company was able to provide de-identified productivity information for 4,873 cane cutters hired for the 2015–2016 harvest.

#### Wet bulb globe temperature

The primary predictor of interest was wet bulb globe temperature (WBGT) which is a composite measure that combines ambient temperature, humidity, solar radiation, and wind speed and is commonly used in studies where workers are exposed to direct sunlight [7,8,11]. Meteorological observations from the Cengicaña weather station (14.33° N, 91.05° W, 300 meters above sea level) were used to compute the WBGT at 15-minute intervals. The observations required for the WBGT computations included ambient near-surface air temperature (*T*_*a*_), relative humidity (*RH*), incident solar radiation at the surface (*S*), and wind speed (*U*). The WBGT is calculated for outdoor conditions with a solar load using the equation proposed by OSHA [26]:

$$WBGT\;=\;{0.7T}_{wb}+{0.2T}_{g}+{0.1T}_{a},$$ (1)

Where T_*wb*_ is the wet bulb temperature, *T*_*g*_ is the black globe temperature, as measured by a thermometer inserted in the center of a copper globe painted matte black [27], and *T*_*a*_ is the station-observed ambient air temperature as noted above (all temperature units are in °C). *T*_*wb*_ is estimated from *T*_*a*_ and *RH* using the empirical equation of Stull, 2011 [28], which has a mean absolute error < 0.3°C for the range of temperatures encountered in this study. It is noteworthy that the method of Stull, 2011 [28] yields the psychrometric web bulb temperature (as opposed to the natural wet bulb temperature) which is required for the *WBGT* approach we employ that follows Dimiceli et al., 2011 [27], and Weatherly and Rosenbaum, 2017 [29], as described next. While *T*_*g*_ can be directly measured with proper instrumentation, it is not common to do so because of the expense, and therefore *T*_*g*_ is usually estimated using other weather variables. *T*_*g*_ was computed following the physically-based formulation of Dimiceli et al., 2011 [27], which requires observed *T*_*a*_, *RH*, *S*, *U*, and the station coordinates as inputs. Several parameters are also required; we employed identical parameters to those used by Weatherly and Rosenbaum, 2017 [29], who also used the Dimiceli et al., 2011 [28] equations. Three additional modifications were made. First, we directly used the observed incident solar radiation (*S*), whereas Weatherly and Rosenbaum, 2017 [29] estimated *S* because they did not have measurements. Second, we found that the equation for *T*_*g*_ becomes unstable at very low solar zenith angles—i.e., at sunrise each day when the sun is low on the horizon and the solar zenith angle is between 85–90°–and therefore solar zenith angles > 85° were set to 85°. Third, we set a minimum wind speed, *U*, of 1 m s^-1^ following the approach of Lemke and Kjellstrom, 2012 [30], which accounts for the fact that airflow will rarely be zero relative to the skin of humans working outdoors.

For each day, we computed the distribution of the 15-minute WBGT measurements during the working hours of 07:00 to 17:00. We then selected the 95^th^ percentile value as the WBGT_95_ measurement for that day. This approach allowed us to examine high values of daily WBGT exposure that may be the most important contributors to worker heat-related health effects [14] while protecting against values so high that they may be considered outliers. Additionally, we calculated the mean work-shift WBGT (WBGT_mean_) that workers were exposed to during typical working hours (07:00–17:00).

#### Kidney function

We were interested in determining whether kidney function at the start of the harvest modified the relationship between WBGT exposure and worker productivity. Serum creatinine was used to calculate the estimated Glomerular Filtration Rate (eGFR) using the Chronic Kidney Disease Epidemiology Collaboration (CKD-EPI) equation [31] for each worker. All workers were male and race was considered “non-black” for all participants in the CKD-EPI equation. We dichotomized workers based on eGFR at or above 60 ml/min/1.73 m^2^, defined as having functioning kidneys, and below 60 ml/min/1.73 m^2^, defined in this analysis as having impaired kidney function, since this is a commonly agreed upon cut-off for defining CKD Stage 3 [32].

### Statistical analysis

Building upon studies that have shown temperature and health do not follow a linear trend [33, 34], we chose to use distributed lag non-linear models as a flexible way to model the relationship between temperature exposure and productivity. Additionally, distributed lag non-linear models allowed us to also address the potentially important lagged effect of temperature exposure [35, 36] by estimating the effect on productivity in each future day following an exposure to increased WBGT.

When the effect of an exposure on the outcome may not be limited to the period when it is observed, lags can be used to determine the effect of the exposure at different times over the course of an event [33]. To model the initial exposure as well as the lag dimension, distributed lag non-linear models can be used [33]. Distributed lag non-linear models are based on the concept of a cross-basis, which is the joint modeling of two bases. In the framework of a distributed lag non-linear model, the two bases that the cross-basis is comprised of represent the exposure, as well as the lag. The cross-basis is then used as a predictor in a typical regression model to relate the exposure and lags simultaneously to the outcome. The complete theoretical framework for distributed lag non-linear models can be found in Gasparrini, et al 2010 [33].

For each of our outcomes, WBGT_95_ and WBGT_mean_, fit a linear regression model for the relationship between the cross-basis of temperature exposure and lagged temperature exposure, with average tons produced. Each model was then stratified on eGFR categories (<60 ml/min/1.73 m^2^ vs. ≥60 ml/min/1.73 m^2^).

To calculate the cross-basis, the relationship between temperature exposure and average daily tons produced was modelled with natural splines with four degrees of freedom. The degrees of freedom were selected by plotting the smoothed relationship between WBGT and average daily tons while varying the degrees of freedom. To determine the number of lags to be included in the analysis, we examined the cross-correlation between both WBGT_95_ and WBGT_mean_ with average daily tons. The cross-correlation suggested that five lags were appropriate. Five lags were also a theoretically appropriate number of lags to use, since a typical work week is six days long. We modeled the lagged relationship of temperature exposure and average daily tons produced with three strata. The effect on average tons produced was assumed constant within each stratum. The dlnm package [37] in R version 3.4.3 [38] was used to fit these models.

As a sensitivity analysis we re-ran the distributed lag non-linear models with the exclusion of the months November and May. This allowed us to account for effects that may be due in part to the seasonality of productivity, where production is naturally slower in the start of the season when they are in the acclimatization phase, as well as the end of the season when there is less sugarcane to harvest.

Cox proportional hazard models were run to assess differences in worker attrition between eGFR categories. Proportional hazard assumptions were visually checked using the log-negative log plot of the Kaplan Meier estimated survival curves. The dataset was stratified for those who dropped out prior to week 11 and those who dropped out during or after week 11 due to violations of the proportional hazards assumption. To account for the potential confounding effect of age, age was included as a continuous covariate in the Cox proportional hazard models. To perform demographic comparisons between eGFR categories (<60 ml/min/1.73 m^2^ vs. ≥60 ml/min/1.73 m^2^), t-Tests with Satterthwaite approximations were used for continuous variables and Chi-square tests were used for categorical variables. These analyses were done using SAS version 9.4 (Cary, NC).

## Results

There were 4,095 (80%) hired cane cutters who met inclusion criteria. All cane cutters who met inclusion criteria were male. As shown in Table 1, 77 (2%) of the hired cohort had impaired kidney function at the time of pre-employment screening. The average worker was 30 years (SD: 9), had worked 7 previous harvests (SD: 7), and had a Body Mass Index (BMI) of 23 (SD: 3). The average amount of sugarcane cut per day was 5.9 tons (SD: 3.4). Workers with impaired kidneys were unable to cut as much sugarcane per day compared to those with higher kidney function; this amounted to an average of 5.62 (SD: 2.67) tons per day compared to 5.76 (SD: 2.72) tons per day, respectively. Workers with impaired kidney function were significantly less likely to complete the harvest, 42% (N: 32) of workers left before the end of the season compared to 25% (N: 1,019) (Table 1).

**Table 1:** Univariate relationship between kidney function at the start of the 2015–2016 harvest and variables of interest.

	eGFR < 60 (N = 77) Mean (SD) or %	eGFR ≥ 60 (N = 4018) Mean (SD) or %	p-value
Age	38.37 (11.29)	29.78 (9.16)	<0.0001
Body Mass Index	23.10 (3.00)	23.11 (2.92)	0.9729
Previous harvests worked	10.07 (10.26)	7.03 (6.66)	0.0116
Average tons cut per day worked	5.62 (2.67)	5.76 (2.72)	<0.0001
Attrition	32 (41.56%)	1019 (25.36%)	0.0013

**Fig 2:**
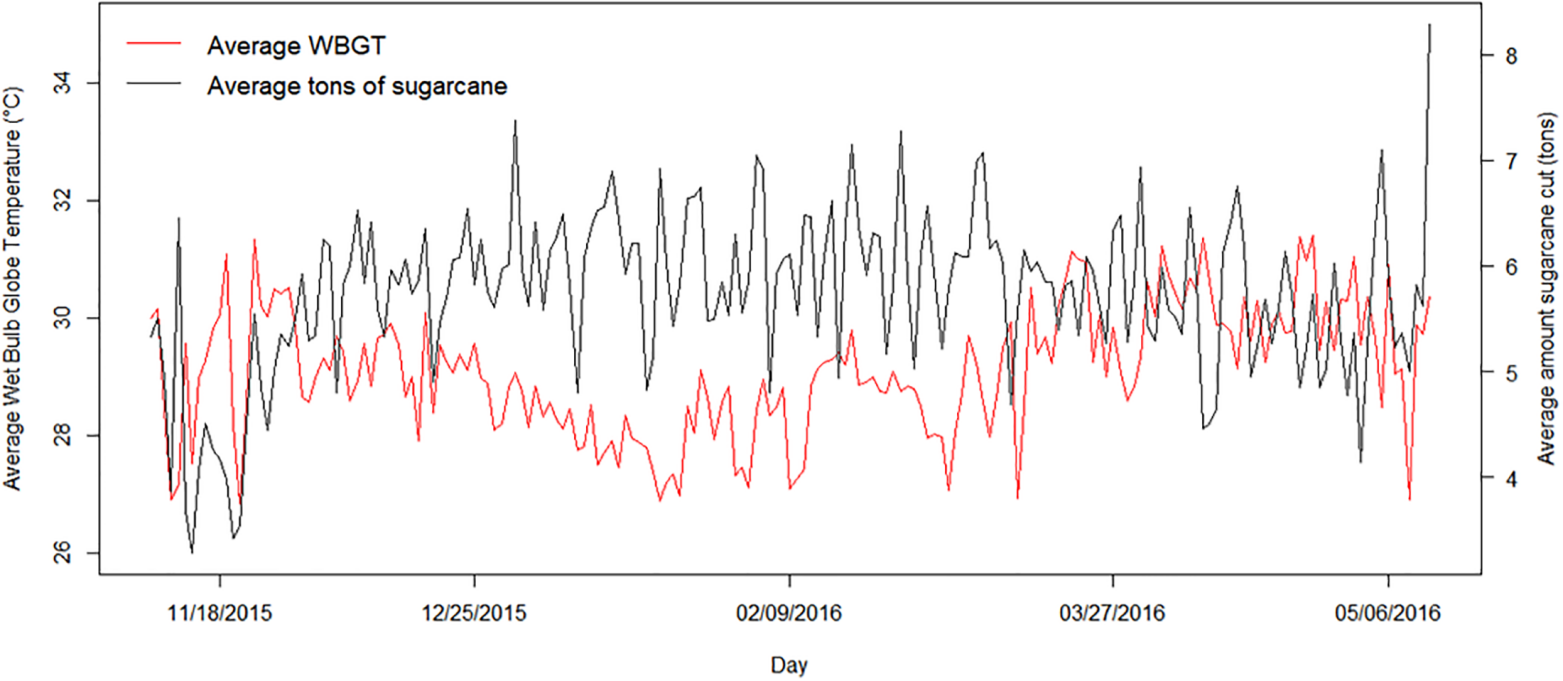
The daily mean WBGT along with average daily tons produced, through the 2015–2016 sugarcane harvest.

The median WBGT_95_ was 32.13°C (IQR: 1.84°C; Range: 29.11°C to 35.94°C). The median WBGT_mean_ was 29.07°C (IQR: 1.42°C; Range: 26.84°C to 31.42°C). The maximum values WBGT_95_ occurred in November. Temperatures appear to decline from December through January and begin to rise again through March, at which time they appear to stabilize through the end of the harvest (Fig 2).

**Fig 3:**
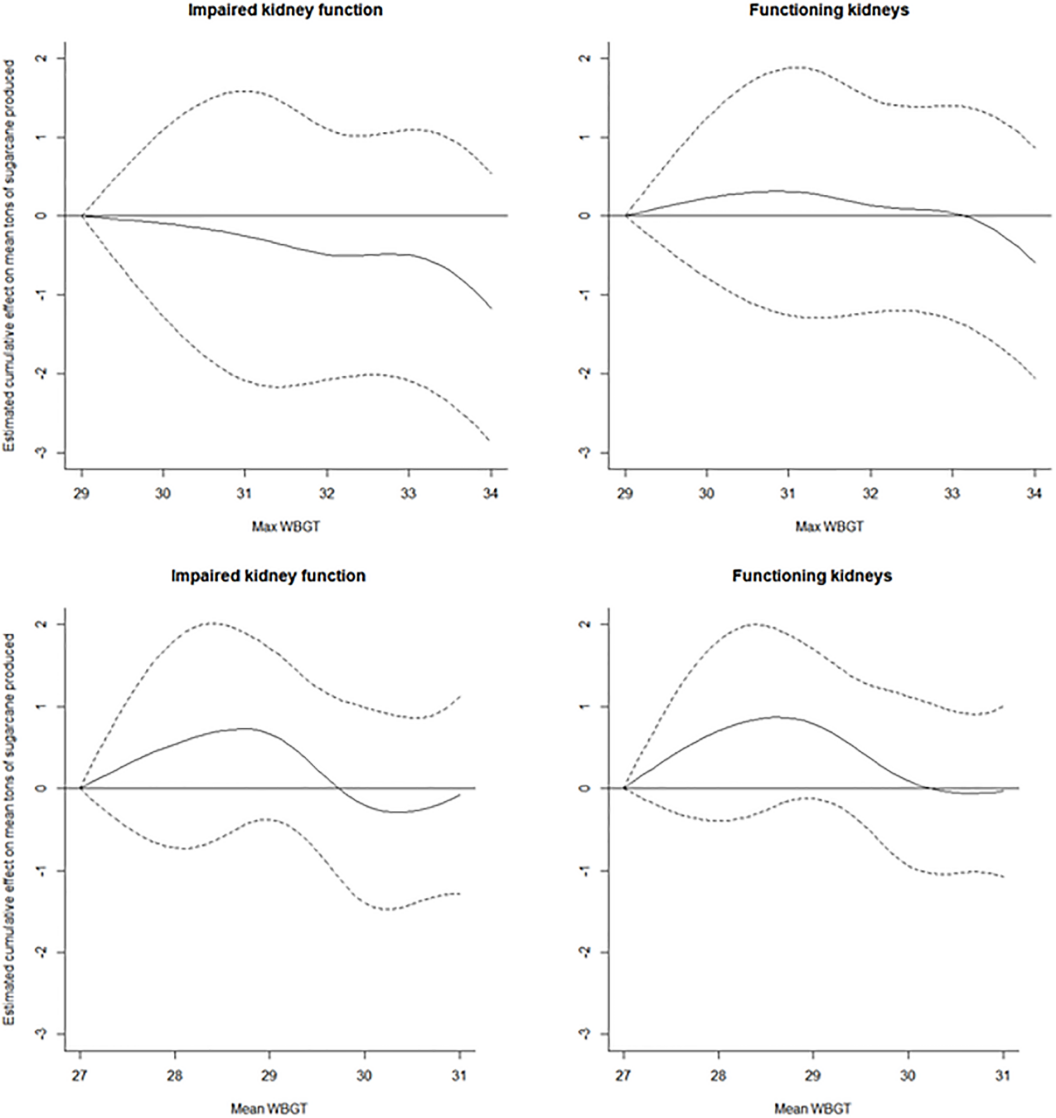
Cumulative association and 95% confidence interval over five-day lag between temperature and average daily tons produced. (Top) Temperature was defined using the 95^th^ percentile of WBGT during the work-shift with a reference of 29°C (Bottom) Temperature was defined using the mean work-shift WBGT with a reference 27°C. (Left) Impaired kidney function: eGFR < 60 ml/min/1.73 m^2^. (Right) Functioning kidneys: eGFR ≥ 60 ml/min/1.73 m^2^.

The use of a distributed lag non-linear model allowed us to assess the cumulative effect on productivity over future days following exposure to an increased WBGT [33]. In this case, lags up to five days were included in the model, thus providing us with the overall cumulative effect on the average productivity over the next five days given an increase in WBGT exposure (Fig 3, Tables 2 and 3). The cumulative effect on tons of sugarcane cut for workers with impaired kidney function who experienced exposure to a WBGT_95_ of 34°C is estimated to be a loss of 1.16 (95% confidence interval (CI): -2.87, 0.54) tons over the next five days compared to if they were exposed to a WBGT_95_ of 29°C. The estimated cumulative effect on tons of sugarcane cut by workers with functioning kidneys was 0.59 tons (95% CI: -2.05, 0.87) less under the same circumstances. The general trend for WBGT_95_ was that as the temperature of exposure increased, the cumulative reduction in the amount of sugarcane cut over the next five days was more pronounced. The cumulative effect on productivity in the WBGT_95_ model was greater for those with impaired kidney function, although not statistically different than those with functioning kidneys. There was no evidence to suggest that exposure to increased WBGT_mean_ influenced cumulative productivity for either group. Lag specific estimates for a range of temperature exposures can be found in S1 Fig.

**Table 2:** Overall cumulative association between WBGT_95_ and tons of sugarcane produced accounting for five day lagged effects. All estimates are calculated relative to a reference WBGT of 29°C.

95th percentile WBGT exposure	All workers Cumulative effect (95% CI)	eGFR < 60 Cumulative effect (95% CI)	eGFR ≥ 60 Cumulative effect (95% CI)
30	0.22 (-0.79, 1.24)	-0.09 (-1.28, 1.09)	0.23 (-0.78, 1.24)
31	0.30 (-1.27, 1.87)	-0.25 (-2.08, 1.58)	0.31 (-1.26, 1.88)
32	0.13 (-1.24, 1.49)	-0.49 (-2.08, 1.10)	0.14 (-1.22, 1.50)
33	0.03 (-1.33, 1.39)	-0.50 (-2.08, 1.09)	0.04 (-1.32, 1.40)
34	-0.60 (-2.06, 0.86)	-1.16 (-2.87, 0.54)	-0.59 (-2.05, 0.87)

**Table 3:** Overall cumulative association between WBGT_mean_ and tons of sugarcane produced accounting for five day lagged effects. All estimates are calculated relative to a reference WBGT of 27°C.

Mean WBGT exposure	All workers Cumulative effect (95% CI)	eGFR < 60 Cumulative effect (95% CI)	eGFR ≥ 60 Cumulative effect (95% CI)
28	0.71 (-0.40, 1.81)	0.55 (-0.72, 1.82)	0.71 (-0.39, 1.81)
29	0.79 (-0.12, 1.70)	0.67 (-0.38, 1.72)	0.79 (-0.12, 1.70)
30	0.09 (-0.95, 1.12)	-0.20 (-1.39, 0.99)	0.09 (-0.94, 1.13)
31	-0.03 (-1.08, 1.01)	-0.08 (-1.29, 1.12)	-0.03 (-1.08, 1.01)

**Fig 4:**
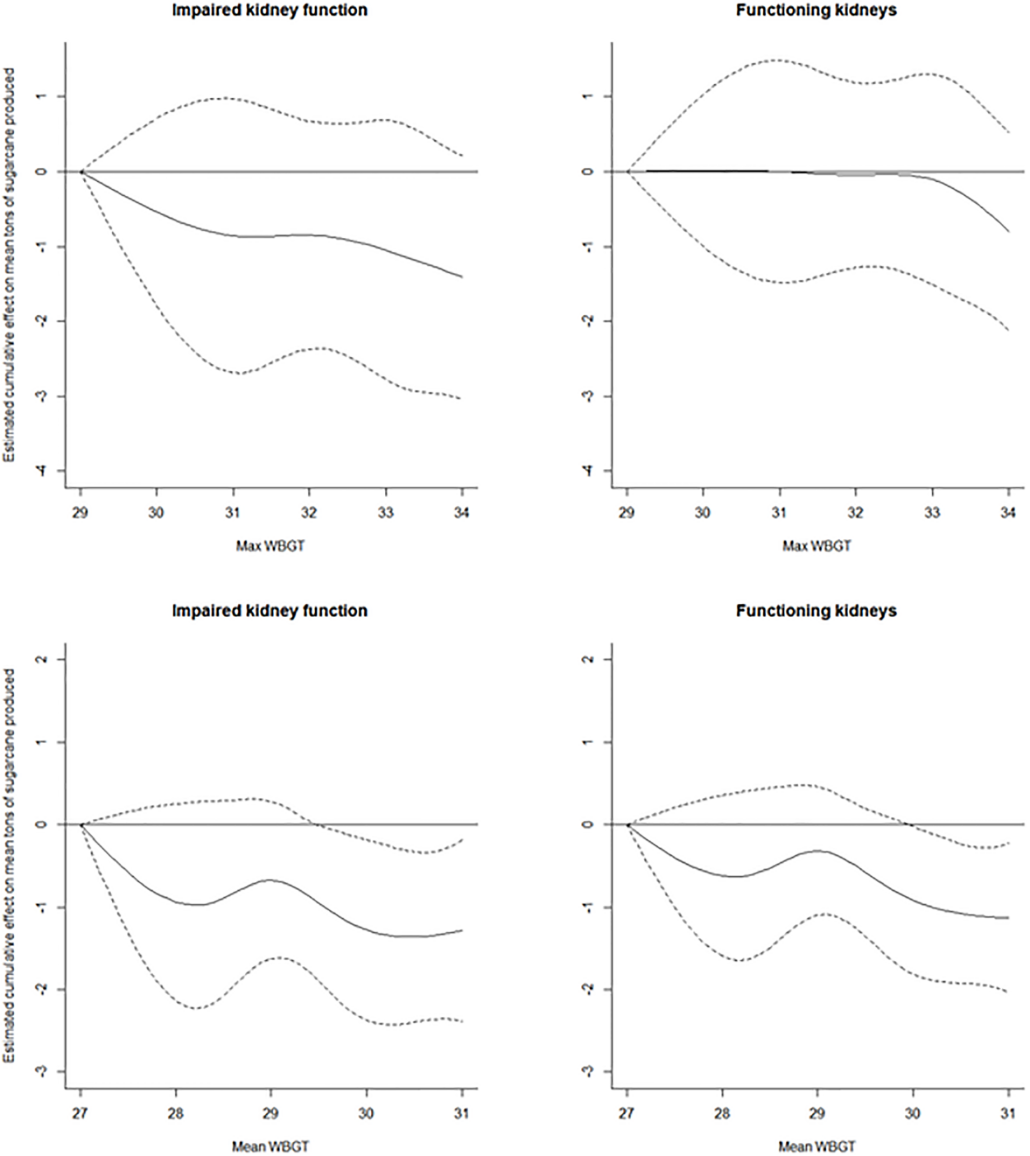
Cumulative association and 95% confidence interval over five-day lag between temperature and average daily tons produced. November and May removed. (Top) Temperature was defined using the 95^th^ percentile of WBGT during the work-shift with a reference of 29°C (Bottom) Temperature was defined using the mean work-shift WBGT with a reference 27°C. (Left) Impaired kidney function: eGFR < 60 ml/min/1.73 m^2^. (Right) Functioning kidneys: eGFR ≥ 60 ml/min/1.73 m^2^.

Periods of lower productivity occurred at the start of the season during the acclimatization phase, as well as the end of the season when production is wrapping up. These natural periods of lower productivity corresponded with increased temperatures (Fig 2). To assess the stability of our estimates, we performed the analysis with data from November and May removed. Interestingly, the effect on productivity of increased WBGT exposure became more pronounced in this analysis (Fig 4, Tables 4 and 5). For those with impaired kidney function, the cumulative effect on productivity was estimated to be 1.41 tons (95% CI: -3.04, 0.22) less over five days for an exposure to WBGT_95_ of 34°C compared to an exposure of 29°C. For those with functioning kidneys, the cumulative effect on productivity was estimated to be 0.80 tons (95% CI: -2.12, 0.52) less under the same circumstances. The most noticeable difference was in the estimates of the effect of WBGT_mean_ on cumulative productivity. For both the impaired and functioning groups, there was a statistically significant effect of exposure to WBGT_mean_ of 31°C compared to 27°C on cumulative productivity. The impaired group was estimated to produce 1.28 tons (95% CI: -2.38, -0.18) less over five days, while the functioning group was estimated to produce 1.13 tons (95% CI: -2.03, -0.23) less.

**Table 4:** Overall cumulative association between WBGT_95_ and tons of sugarcane produced accounting for five day lagged effects. All estimates are calculated relative to a reference WBGT of 29°C. November and May removed.

95th percentile WBGT exposure	All workers Cumulative effect (95% CI)	eGFR < 60 Cumulative effect (95% CI)	eGFR ≥ 60 Cumulative effect (95% CI)
30	0.01 (-1.01, 1.02)	-0.54 (-1.79, 0.71)	0.02 (-1.00, 1.03)
31	-0.02 (-1.50, 1.47)	-0.86 (-2.69, 0.97)	0.00 (-1.48, 1.48)
32	-0.06 (-1.30, 1.18)	-0.85 (-2.37, 0.68)	-0.05 (-1.28, 1.19)
33	-0.12 (-1.53, 1.28)	-1.05 (-2.78, 0.68)	-0.11 (-1.51, 1.30)
34	-0.81 (-2.13, 0.51)	-1.41 (-3.04, 0.22)	-0.80 (-2.12, 0.52)

**Table 5:** Overall cumulative association between WBGT_mean_ and tons of sugarcane produced accounting for five day lagged effects. All estimates are calculated relative to a reference WBGT of 27°C. November and May removed.

Mean WBGT exposure	All workers Cumulative effect (95% CI)	eGFR < 60 Cumulative effect (95% CI)	eGFR ≥ 60 Cumulative effect (95% CI)
28	-0.62 (-1.60, 0.36)	-0.94 (-2.13, 0.26)	-0.62 (-1.60, 0.36)
29	-0.32 (-1.11, 0.46)	-0.67 (-1.62, 0.28)	-0.32 (-1.10, 0.46)
30	-0.92 (-1.82, -0.03)	-1.28 (-2.37, -0.18)	-0.92 (-1.81, -0.02)
31	-1.13 (-2.03, -0.23)	-1.28 (-2.38, -0.18)	-1.13 (-2.03, -0.23)

**Fig 5:**
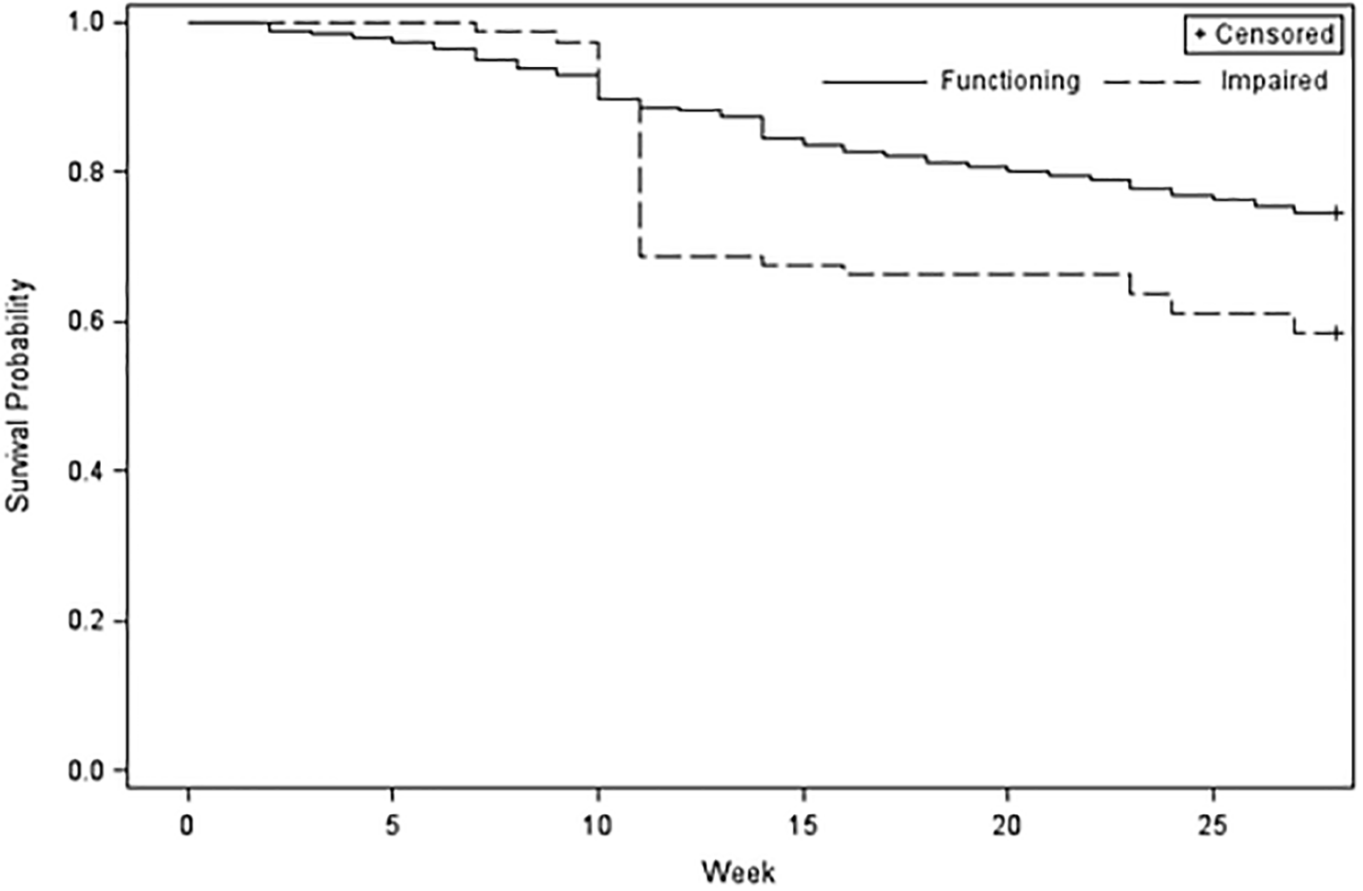
Kaplan Meier estimated survival curves for attrition stratified by kidney function. (Impaired kidney function) eGFR < 60 ml/min/1.73 m^2^ represented by the dashed lined. (Functioning kidneys) eGFR ≥ 60 ml/min/1.73 m^2^ represented by the solid line.

Productivity can be adversely affected in two ways: reduced daily tons produced and premature workforce attrition. We observed a notable difference in job attrition between those with impaired kidney function and those with functioning kidneys (Fig 5). Table 6 summarizes the estimated hazard ratios for those who left the workforce before week 11 of the 28-week harvest compared to those who left during week 11 or later. Workers who started the season with kidney impairment were more likely to leave the workforce before the harvest season was over, even after controlling for age. There was no effect of kidney function on attrition prior to week 11. However, kidney function was related to attrition on or after week 11. The likelihood of leaving the workforce during week 11 or later for those with impaired kidney function was 2.92 (95% CI: 1.88, 4.32) the likelihood for those with functioning kidneys after adjusting for age.

**Table 6:** Age adjusted hazard ratio estimates for leaving the workforce before the end of the season (attrition) before and after week 11.

	eGFR < 60 versus eGFR ≥ 60	
Hazard	Estimate	95% CI
Attrition prior to week 11	0.56	(0.24, 1.11)
Attrition during or after week 11	2.92	(1.88, 4.32)

## Discussion

In this study, we evaluated the health and daily productivity of 4,095 sugarcane workers in Guatemala to address the hypothesis that there would be a direct inverse relationship between temperature exposure and the amount of sugarcane produced with the greatest impact being on those with impaired kidney function at the start of the harvest. While not definitive, our analyses suggest that increases in daily maximum WBGT negatively affected productivity over the next five days. This was more apparent in the impaired kidney function group, although not statistically significant, when compared to those with an eGFR > 60 ml/min/1.73 m^2^ at the start of the harvest. This study provides initial field evidence for the link between impaired kidney function, increased heat exposure, and agricultural worker productivity.

Our data suggest that as exposure to heat increases, agricultural production may decrease. This supports findings from other studies on heat and productivity [4,10]. Other investigators have estimated that there is a 0.57% reduction in direct work time [8] and 5% reduction in work output of rice harvesters [7] with increases in WBGT. Conversely, one study found only limited evidence of an effect of WBGT on declined tree fruit harvest [11] although the authors point out that it was conducted in a more moderate climate than that found in Central America. Estimates show that in parts of Central and South America, up to 10–15% of annual daylight hours are so hot that productivity is lost [14]. Under heavy work conditions, regular rest is recommended when the WBGT exceeds 26°C [14]. There is evidence that workers with impaired kidney function cut less sugarcane per day than do those with functioning kidneys. Our data suggest that the productivity of workers with impaired kidney function may be more greatly impacted by exposure to increase heat extremes. It is important to consider the role of acute and chronic health conditions on worker productivity in future projections of climate’s effect on worker productivity.

Additionally, we have shown that workers with impaired kidney function have more than twice the risk of not finishing a harvest, resulting in fewer days worked. This is consistent with the estimate that global labor capacity has decreased 5.3% from 2000 to 2016 due to temperature change [1]. Productivity, economic output, pay, and family income are all reduced as a result of an injured worker’s natural inclination to slow down work or limit working hours [14]. In low- and middle- income countries that are dependent on manual labor, the health and welfare of workers are of paramount importance for sustained industrial growth [10]. Thus, our data illustrate the inherent conflict between preserving health and maintaining productivity that workers and employers must address [14]. This challenge is particularly urgent in the case of CKDu, in light of evidence implicating high energy expenditure during temperature extremes as a potential contributor to kidney insufficiency [12,19,39].

Our data demonstrate that lost productivity in agricultural workers with impaired kidney function is reflected by the likelihood that workers will drop out of the workforce as well as how much of a commodity is produced. These findings, taken in context with the body of evidence suggesting a relationship between work in hot climates and the epidemic of CKDu [2,12,39] leads us to propose a ‘two-hit’ hypothesis for how heat exposures impact worker productivity and health. Multiple studies have implicated high intensity work in hot climates as contributors to the global epidemic of CKDu [2]. In turn, we have provided evidence suggesting that sugarcane workers with kidney insufficiency are more vulnerable, suffering greater impact on their productivity and employment as temperature exposure increases. Our data provide initial empirical support that climate can directly impact commodity production [40]. Taken together, published models and our data suggest that the extent of food insecurity is being underestimated when climate models of our world’s food supply neglect heat’s effect on worker health and workforce sustainability. Our findings reinforce the need to better understand the role of heat in the development of both acute and chronic health conditions, such as chronic kidney disease, especially as the global burden of kidney disease and disability continues to rise [21].

This study carries a few limitations. We evaluated the effect of heat on productivity of only hired workers during a single season in a single large agribusiness, thus results are not necessarily generalizable to all agricultural workers. Measurements of WBGT were calculated using data from a weather station in proximity to the fields where the sugarcane cutters were working. The station is at a higher altitude than most of the fields, thus providing us with relatively lower temperature readings. While this provided us with a conservative estimate of WBGT, we acknowledge that the estimated impact of heat on productivity is likely an underestimate. Additionally, we related average daily productivity to aggregate measures of WBGT during work hours. By doing so we were limited in our ability to determine how heat progression throughout the work day impacted productivity as well as our ability to determine the effect of temperature exposure outside of work hours. As shown in Fig 2, week 11 is around the time that the seasonal temperatures begin to rise. Consequently, we have fewer observations on the productivity of impaired kidney function workers at higher values of WBGT, making estimates for those workers at increased temperatures less stable and making relationships between temperature exposure and productivity harder to detect for those workers. In this analysis we did not temporally decompose long-term trends in productivity, which may explain some of the observed association between increased temperature exposure and decreased productivity.

In conclusion, this study provides support for connecting heat extremes to productivity of agricultural workers, which is more apparent in those experiencing impaired kidney function. In light of the rise in the global burden of non-vector borne, chronic illness [21,41], the epidemic of work-related chronic illnesses that have been related to heat, including CKDu [2], and evidence of chronic kidney disease in Central American agricultural communities [15,16], we call for further research and interventions that help address this public health crisis. There is an urgent need to identify how these relationships impact productivity, labor loss, income, food accessibility, and most importantly, health and economic outcome disparities among vulnerable workers and their families.

## Supporting information

S1 Fig Lag specific effects on average daily tons produced estimated at three different WBGT exposures. (Top) Temperature was defined using the 95^th^ percentile of WBGT during the work-shift with a reference of 29°C (Bottom) Temperature was defined using the mean work-shift WBGT with a reference 27°C. (Left) Impaired kidney function: eGFR < 60 ml/min/1.73 m^2^. (Right) Functioning kidneys: eGFR ≥ 60 ml/min/1.73 m^2^.
(PDF)
