# Supplementary figures and images for: The impact of heat and impaired kidney function on productivity of Guatemalan sugarcane workers
Source: PLoS One. 2018 Oct 5;13(10):e0205181. doi: 10.1371/journal.pone.0205181 (PMC6173423; doi:10.1371/journal.pone.0205181)

## WBGT<sub>95</sub>

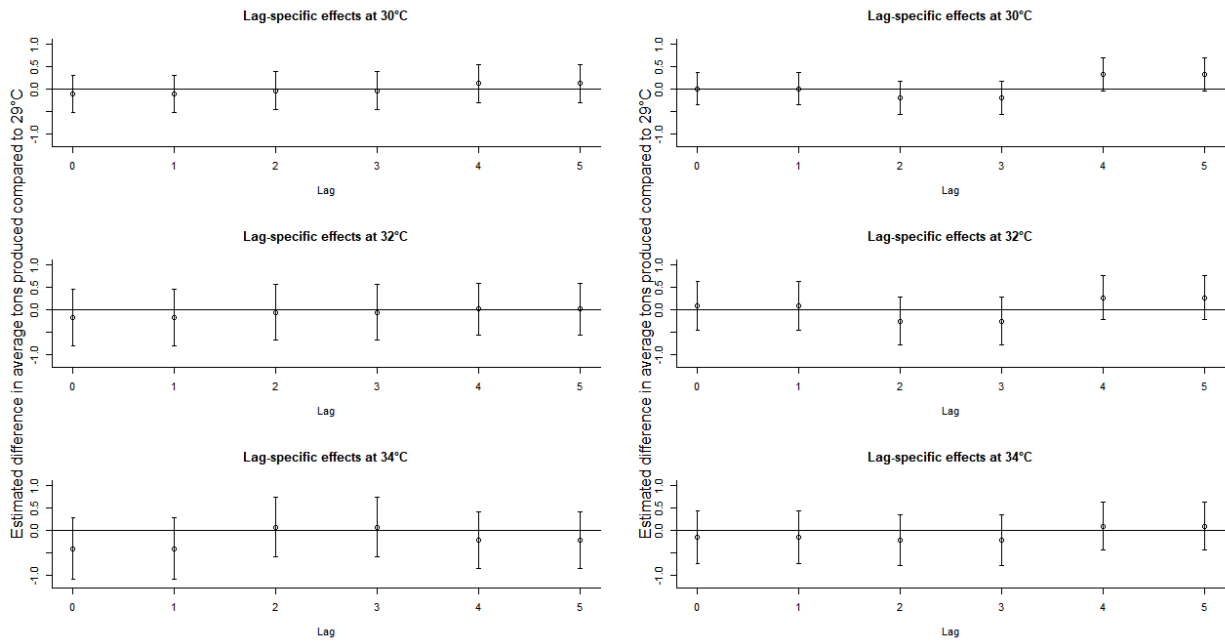

## WBGT<sub>mean</sub>

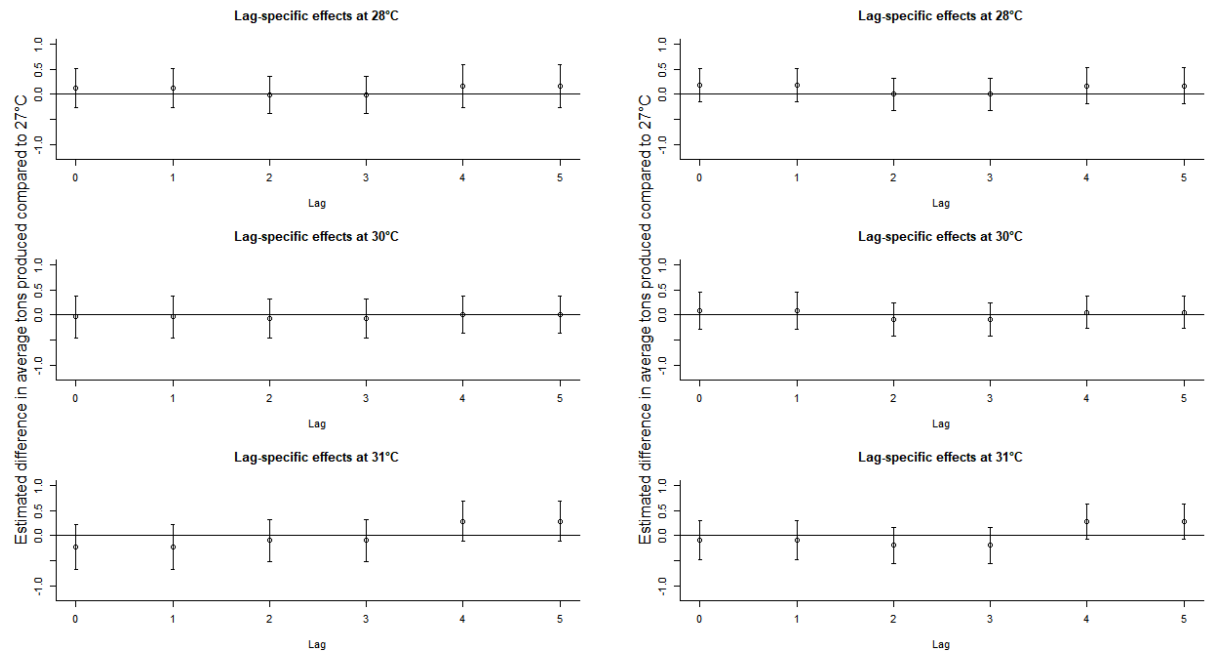

Supplement: S1 Fig — (Top) Temperature was defined using the 95th percentile of WBGT during the work-shift with a reference of 29°C (Bottom) Temperature was defined using the mean work-shift WBGT with a reference 27°C. (Left) Impaired kidney function: eGFR < 60 ml/min/1.73 m2. (Right) Functioning kidneys: eGFR ≥ 60 ml/min/1.73 m2. (PDF) [file pone.0205181.s001.pdf]
